# Phylogenetics of Tribe Collabieae (Orchidaceae, Epidendroideae) Based on Four Chloroplast Genes with Morphological Appraisal

**DOI:** 10.1371/journal.pone.0087625

**Published:** 2014-01-31

**Authors:** Xiao-Guo Xiang, Wei-Tao Jin, De-Zhu Li, André Schuiteman, Wei-Chang Huang, Jian-Wu Li, Xiao-Hua Jin, Zhen-Yu Li

**Affiliations:** 1 State Key Laboratory of Systematic and Evolutionary Botany, Institute of Botany, Chinese Academy of Sciences, Beijing, China; 2 Key Laboratory of Biodiversity and Biogeography, Kunming Institute of Botany, Chinese Academy of Sciences, Kunming, Yunnan, China; 3 Herbarium, Library, Art and Archives Directorate, Royal Botanical Gardens, Kew, Richmond, Surrey, United Kingdom; 4 Shanghai Chenshan Botanical Garden, Songjiang, Shanghai, China; 5 Herbarium, Xishuanbanna Tropical Botanical Garden, Chinese Academy of Sciences, Menglun Township, Mengla County, Yunnan, China; George Washington University, United States of America

## Abstract

Collabieae (Orchidaceae) is a long neglected tribe with confusing tribal and generic delimitation and little-understood phylogenetic relationships. Using plastid *matK*, *psaB*, *rbcL*, and *trnH*-*psbA* DNA sequences and morphological evidence, the phylogenetic relationships within the tribe Collabieae were assessed as a basis for revising their tribal and generic delimitation. Collabieae (including the previously misplaced mycoheterotrophic *Risleya*) is supported as monophyletic and nested within a superclade that also includes Epidendreae, Podochileae, Cymbidieae and Vandeae. *Risleya* is nested in Collabiinae and sister to *Chrysoglossum*, a relationship which, despite their great vegetative differences, is supported by floral characters. *Ania* is a distinct genus supported by both morphological and molecular evidence, while redefined *Tainia* includes *Nephelaphyllum* and *Mischobulbum*. *Calanthe* is paraphyletic and consists four clades; the genera *Gastrorchis*, *Phaius* and *Cephalantheropsis* should be subsumed within *Calanthe*. *Calanthe* sect. *Ghiesbreghtia* is nested within sect. *Calanthe*, to which the disputed *Calanthe delavayi* belongs as well. Our results indicate that, in Collabieae, habit evolved from being epiphytic to terrestrial.

## Background

The family of Orchidaceae is one of largest among seed plants, and several classification systems have been proposed to reflect their phylogeny and evolution (such as [1]–[4]). Recent molecular phylogenetic studies have shed new light on the relationships of Orchidaceae from genera to subfamilies (such as [5]–[16]). In Collabieae, as in many orchids, relationships have been traditionally defined based on subjective assessments of morphological characters, and phylogenetic relationships remain to be assessed using molecular data.

Tribe Collabieae, described by Pfitzer [17] based on *Collabium*, is a medium-sized group with about 450–500 species distributed mainly in the Old World tropics with a few species extending into North Temperate Asia and Mesoamerica [18]–[21]. However, Collabieae has not been recognized by most of subsequent authors, and the genera have been included in different tribes. Schlechter [22] established two subtribes, Collabiinae and Phajinae, in the tribe Kerosphaereae. The former included 7 genera, viz., *Chrysoglossum*, *Collabium*, *Diglyphosa*, *Mischobulbum*, *Nephelaphyllum*, *Pilophyllum*, and *Tainia*, while the latter included 13 genera, i.e., *Acanthephippium*, *Ancistrochilus*, *Anthogonium*, *Aulostylis*, *Bletia*, *Calanthe*, *Chysis*, *Ipsea*, *Phaius*, *Pachystoma*, *Spathoglottis*, *Ascotainia*, and *Plocoglottis*, and it was considered to be a synonym of Bletiinae by most subsequent authors. Dressler & Dodson [23] placed Collabiinae and Phajinae in tribe Epidendreae, whereas Holttum [24] placed these two subtribes in two informal suggested groups, the “*Phaius* tribe” and the “*Nephelaphyllum* tribe”. Dressler [3] placed Phajinae and Collabiinae in Arethuseae and Cymbidieae, respectively, then he [4] listed Collabiinae as one of his “misfits and leftover” groups of uncertain systematic position.

Recent results of cladistic analyses of combined DNA sequences have provided some new insights in the systematics of tribe Collabieae. Based on *matK* and *rbcL*, Goldman et al. [25] proposed that *Mischobulbum*, *Nephelaphyllum*, and *Tainia* should be transferred from Bletiinae (in the tribe Arethuseae) to the non-Arethuseae subtribe Collabiinae. Likewise, based on their analyses of ITS, *matK*, and *trnL*-*F*, van den Berg et al. [26] suggested that Collabiinae and Phajinae should be transferred to tribe Collabieae. Chase et al. [6] and Pridgeon et al. [19] tentatively redefined Collabieae to include 18–19 genera, pending future studies.

As currently defined, Collabieae include *Acanthephippium*, *Ancistrochilus*, *Ania*, *Calanthe*, *Cephalantheropsis*, *Collabium*, *Diglyphosa*, *Eriodes*, *Gastrorchis*, *Hancockia*, *Ipsea*, *Mischobulbum*, *Nephelaphyllum*, *Pachystoma*, *Phaius*, *Pilophyllum*, *Plocoglottis*, *Spathoglottis*, and *Tainia* [19] and shows a variety of vegetative and floral variation, such as plants with corms or pseudobulbs of one to several internodes or without storage organs; leaves petiolate or not and conduplicate or convolute; inflorescences lateral or terminal; pollinia varying from 2 through 4 to 8, and being soft or hard; viscidium present or absent. This diversity has led to difficulties in the circumscription of the tribe, as well as its subtribes and genera, and made problematic to infer the systematic position of the tribe among other Orchidaceae [19], [27]. Moreover, previous molecular systematic studies sampled Collabieae only superficially, with just 7 species in 6 genera in van den Berg et al. [26] and 10 species in 8 genera in Goldman et al. [25]. Due to the sparse sampling and/or weak support for Collabieae in previous molecular systematic studies, subtribal and generic delimitation, as well as the phylogenetic position of Collabieae within Orchidaceae, remain unresolved.

Generic delimitation in many genera within Collabieae is often confused and inconsistent across their distribution range. The *Tainia* alliance, including *Ania*, *Hancockia*, *Mischobulbum*, *Nephelaphyllum*, and *Tainia*, is among the typical cases [18], [19], [24], [27]–[30]. Smith [27] included *Mischobulbum* and *Ascotainia* in *Tainia*. Schlechter [22] maintained *Mischobulbum*, *Hancockia*, and *Ascotainia* as separate genera. Gagnepain [31] considered *Ania*, *Mischobulbum*, *Nephelaphyllum*, and *Tainia* as congeneric. Holttum [24] subsumed *Mischobulbum* and *Ania* into *Tainia*. Seidenfaden [29] included *Ania* in *Tainia*, and kept *Nephelaphyllum*, *Mischobulbum*, and *Hancockia* as distinct genera. Turner [15] recognized *Ania*, *Hancockia*, *Mischobulbum*, *Nephelaphyllum* and *Tainia*. Pearce and Cribb [20] likewise maintained *Ania*, *Tainia*, *Mischobulbum*, and *Nephelaphyllum* as distinct genera (*Hancockia* was not included in their treatment). Chen et al. [21], following Pridgeon et al. [19] treated *Ania*, *Mischobulbum* and *Tainia* as congeneric, and kept *Nephelaphyllum* and *Hancockia* as distinct genera.

Here we use DNA sequences of chloroplast genes *rbcL* and *psaB*, pseudogene *matK*, and the *trnH*-*psbA* region, and a broad sample of taxa across the Collabieae and Orchidaceae in order to: i) clarify the circumscription and systematic position of tribe Collabieae in Orchidaceae; ii) infer phylogenetic relationships within Collabieae; iii) elucidate the delimitation of several debatable genera.

## Results

### Sequences characteristics

In this study, 45 DNA sequences of *rbcL*, 45 of *matK*, 33 of *psaB* and 35 of *trnH-psbA* were newly obtained. For each of the regions studied, aligned sequence lengths and other parsimony-related information are given in Table 1. The subfamily-wide matrix comprised 4674 aligned nucleotides of three chloroplast markers combined: *rbcL* (1362 bp), *psaB* (1666 bp), and *matK* (1646 bp).

**Table 1:** Parsimony statistics from phylogenetic analyses of the various datasets.

Data	Taxa	Aligned length	Information sites	TL	CI	RI
Large matrix						
*rbcL*	116	1362	182	857	0.476	0.693
*matK*	133	1646	433	2890	0.468	0.613
*psaB*	94	1666	207	838	0.548	0.679
combined	133	4674	922	4590	0.474	0.604
Reduced matrix						
*rbcL*	51	1343	56	192	0.698	0.787
*matK*	52	1846	208	678	0.723	0.780
*trnH-psbA*	35	1920	39	105	0.810	0.847
*psaB*	32	1666	37	136	0.809	0.798
combined	52	6775	357	2372	0.702	0.739

TL: tree length; CI: Consistence Index; RI: Retention Index.

In the reduced matrix, the aligned *rbcL* was 1343 bp in length, *psaB* was 1666 bp in length; both were without indels. The *matK* pseudogene was 1846 bp in length with 10 indels from 1 bp to 33 bp. The aligned *trnH-psbA* region was 1919 bp in length among Collabieae. We tried to amplify *trnH*-*psbA* from *Risleya atropurpurea*, but failed. It is possible that this region is lacking in *R. atropurpurea*. The combined dataset of four chloroplast markers was 6775 bp, and more than 4.9% of the characters were parsimony-informative (Table 1).

### Subfamily-wide analysis of Epidendroideae

**Figure 1:**
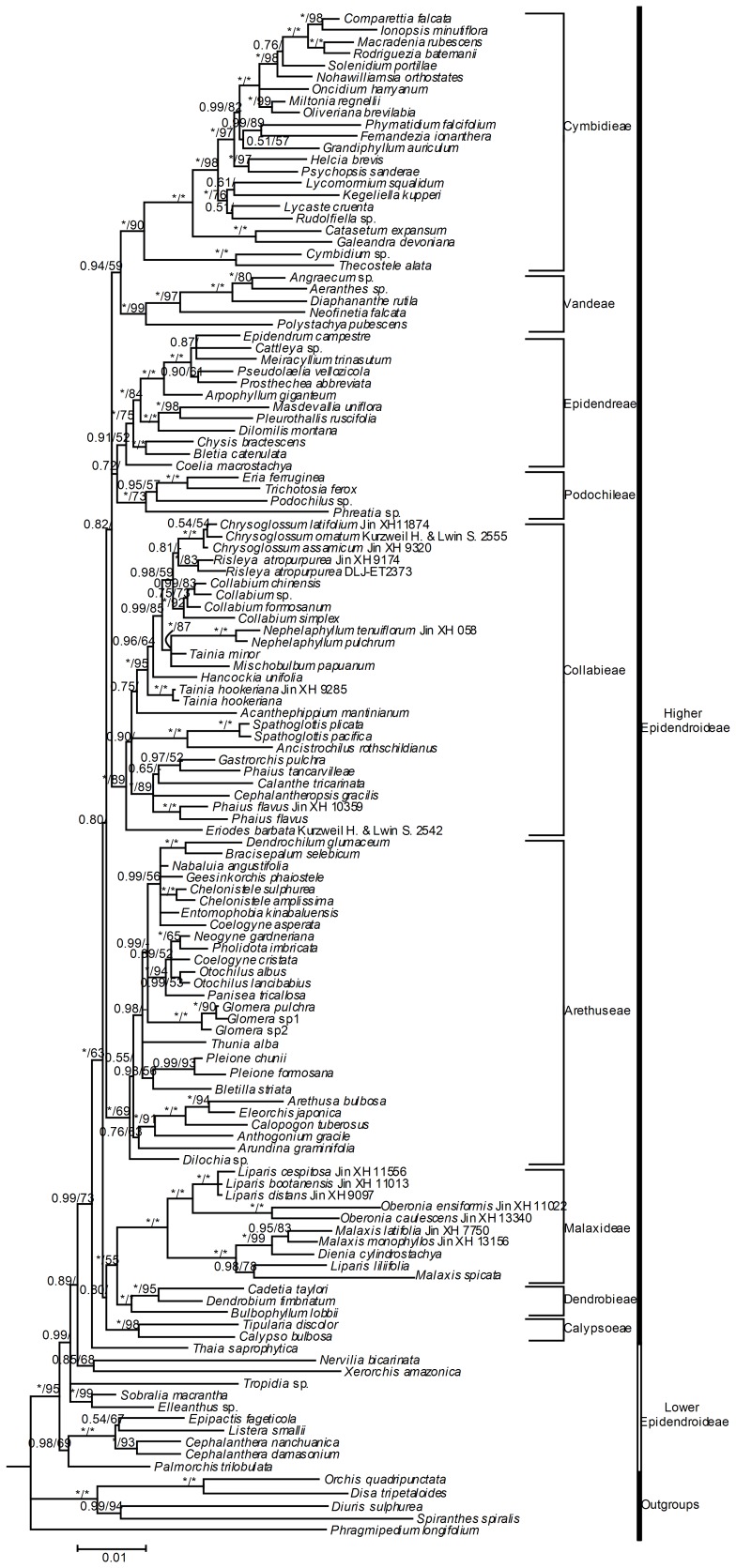
Phylogenetic relationships of subfamily Epidendroideae based on combined *rbcL*, *matK* and *psaB* plastid data. Numbers at the nodes are Bayesian posterior probabilities and bootstrap percentages (>50%), respectively. “-” indicates node is not supported in the analysis. “*” indicates node is with support value 100%.

For the first analysis using the subfamily-wide matrix, under the Bayesian criterion, selection of a partition scheme based on BF favored the P5 partition scheme (see Table 2). The trees generated by BI were congruent with those of MP analysis except for poorly supported nodes along the backbone of the tree (Fig. 1, the MP strict consensus tree is not shown). *Risleya atropurpurea* is identified as a member of tribe Collabieae, not a member of tribe Malaxideae as previously thought [19].

**Table 2:** Summary of Bayesian analyses and 2ln Bayes factor comparisons of partitioning strategies.

Large matrix			Partitioning strategies				
Partitions	Generations	Harmonic mean		P9	P6	P3	P1
P1	3M	−32706.81	P1	1525.52	938.84	904.78	-
P2	3M	−32254.42	P2	620.74	34.06	-	
P3	3M	−32237.39	P3	586.68	-		
P5	3M	−**31944.05**	P5	-			

Reduced matrix			Partitioning strategies				
Partitions	Generations	Harmonic mean		P6	P4	P3	P1
P1	3M	−17207.30	P1	540.14	349.74	310.54	-
P3	3M	−17052.03	P3	229.60	39.20	-	
P4	3M	−17032.43	P4	190.40	-		
P6	3M	−**16937.23**	P6	-	-		

Bayesian parameters are based on combination of two runs.

### Analysis of Collabieae

**Figure 2:**
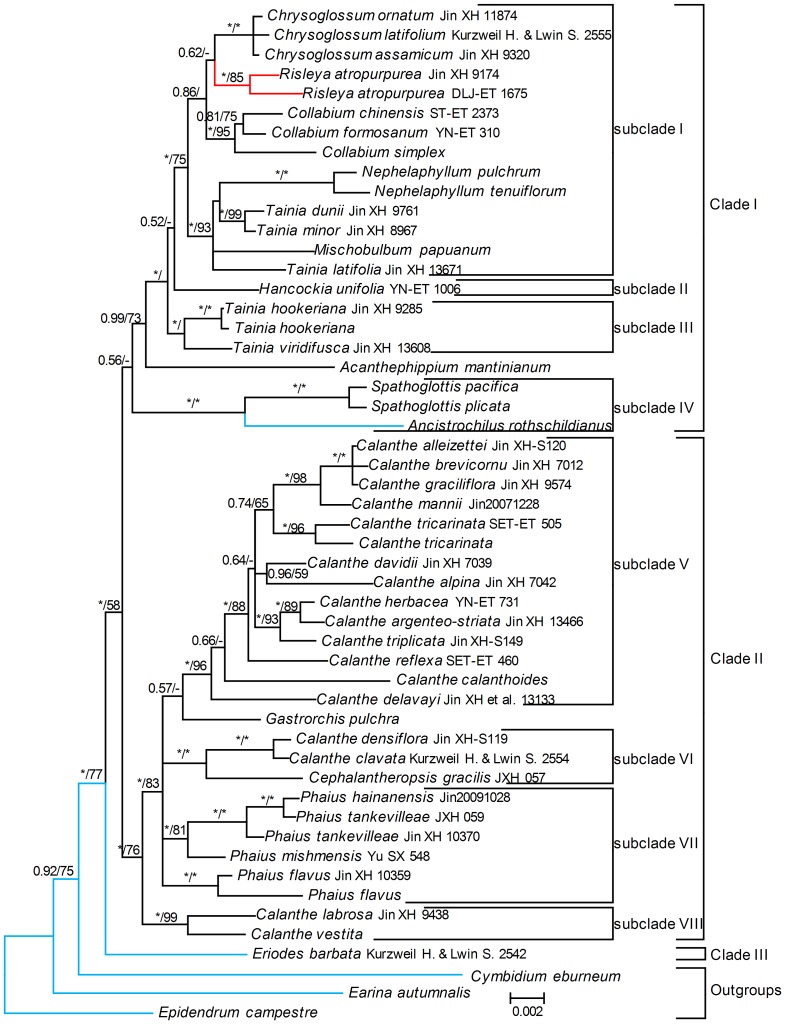
Phylogenetic relationships of the tribe Collabieae based on the four plastid data. Numbers at the nodes are Bayesian posterior probabilities and bootstrap percentages (>50%), respectively. “-” indicates node is not supported in the analysis. “*” indicates node is with support value 100%. Black, red and blue line represent terrestrial, mycoheterotrophic and epiphytic.

For the second analysis with the reduced matrix, P6 was selected as the best-fit partition scheme under the Bayesian criterion (see Table 2). The BI analysis yielded trees with topologies that were consistent with those retrieved by the MP analysis except collapsed nodes (Fig. 2). The parsimony analysis generated 7055 MPTs of 1,175 steps, with a CI of 0.702 and a RI of 0.739. The currently defined Collabieae is subdivided into three clades, of which clade III includes only the monotypic genus *Eriodes* and is sister to two other clades (BS = 77, PP = 1.00).

Clade I consists of sampled genera of Collabiinae, viz., *Acanthephippium*, *Ancistrochilus*, *Chrysoglossum*, *Collabium*, *Hancockia*, *Nephelaphyllum*, *Mischobulbum*, *Spathoglottis*, and *Tainia* plus *Risleya*, a monotypic, mycoheterotrophic genus previously included in tribe Malaxideae [19]. Subclade IV includes the genera *Ancistrochilus* and *Spathoglottis* (BS = 100, PP = 1.00), being sister to other species in Clade I (PP = 0.56). *Acanthephippium* is identified as sister to the other genera in this clade (PP = 1.00), and the remaining genera can be subdivided into 3 subclades: subclade I includes *Chrysoglossum*, *Collabium*, *Mischobulbum*, *Nephelaphyllum*, *Risleya* and five species of *Tainia* (BS = 75, PP = 1.00), subclade II includes monotypic genus *Hancockia*, and subclade III includes two species of *Tainia* (PP = 1.00).

Clade II includes most sampled genera of Phajinae, and is subdivided into five subclades (Figure 2): subclade V includes *Calanthe* sect. *Calanthe*, and *C.* sect. *Ghiesbreghtia* with strong support (BP = 96, PP = 1.00); subclade VI consists of *C.* sect. *Styloglossum* and the genus *Cephalantheropsis* with strong support (BS = 100, PP = 1.00); subclade VII includes *Phaius* without support; subclade VIII includes two species of *Calanthe* subgenus *Preptanthe* with strong support (BP = 99, PP = 1.00), and is sister to subclades V, VI and VII (BP = 74, PP = 1.00).

Cladograms from the morphological data based on BI and MP analyses were poorly resolved (Figure S1). The MP analysis of the morphological data found 51284 equally-shorter trees with a length of 107 steps, a CI of 0.355 and a RI of 0.725. The monophyly of the tribe Collabieae (including *Risleya*) have been recognized in both BI and MP analyses (PP = 0.95, BS = 56).

**Figure 3:**
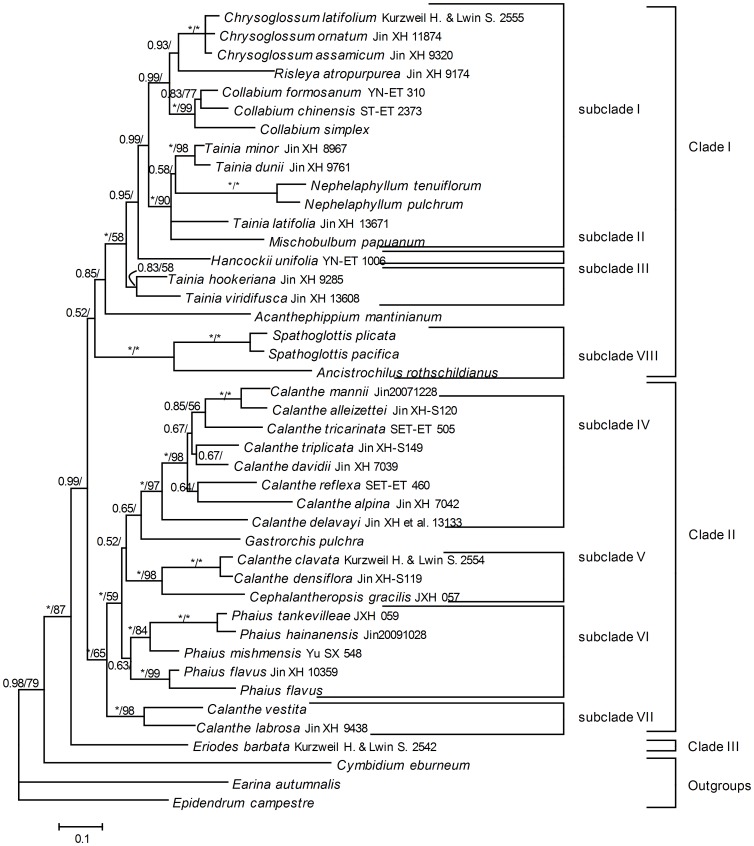
Phylogenetic relationships of Collabieae based on combined data of morphological and molecular evidence. Numbers at the nodes are BI and MP support values (>50%), respectively. “*” represented node with 100% support value.

The combined morphological and molecular data had 6,816 characters, 867 of them variable with 332 (4.9%) parsimony informative. The parsimony analysis generated 360 MPTs of 1,348 steps, with a CI of 0.709 and a RI of 0.691. The topology was consistent to molecular data, except some nodes with higher supporting value (Figure 3).

### The evolution of morphological characters

Our results of the reconstruction of ancestral morphological characters indicated the evolution patterns of morphological characters in Collabieae are complicated. Some morphological characters, such as the presence of rhizodermis, the absence of stipe, are constant or almost so within tribe. Some generic diagnostic morphological characters, such as basal caudicle, column united to base of labellum, non-resupinate flowers, have evolved or were lost several times independently within the tribe or even within same genus. And some morphological characters, such as the inflorescence position, the presence of viscidium, have shifted several times among different states (Figure S2).

## Discussion

### Delimitation of Collabieae

Our results indicate that, as currently delimited, Collabieae, consisting of Collabiinae, Phajinae, and *Eriodes*, is paraphyletic, with *Risleya* having been misplaced in Malaxideae [19]. A redefined Collabieae, including *Risleya*, is strongly supported as monophyletic (Figure 1) and nested within a superclade consisting of Cymbidieae, Epidendreae, Podochileae and Vandeae in Epidendroideae (PP = 0.82). These results differ greatly from most previous taxonomic treatments about Collabieae (such as [3], [4]) but agree well with the suggestions of van den Berg et al. [26] and the tribal circumscription by Pridgeon et al. [19].

The monotypic genus *Risleya* likely has been misunderstood due to its mycoheterotrophic habit and greatly reduced vegetative and floral parts. Although it was placed in Malaxideae by most authors due to the overall floral similarity with some members of Malaxideae [19], [22], [32], [33], it is out of place in Malaxideae by having hairy roots, a rhizome, a cylindrical column, four waxy pollinia in two separate spheroid anther cells (i.e., lacking an anther cap proper), the pollinia attached to a common, large viscidium, a concave stigmatic area under the rostellum, and the elongate rostellum. Chen et al. [33] established a new subtribe to accommodate it. Our morphological examination indicated that some morphological characters, such as hairy roots, waxy pollinia with viscidium, and elongate rostellum, support *Risleya* as a member of Collabieae.

Pridgeon et al. [19] defined Collabieae based on several morphological characters. Our results of morphological characters reconstruction and morphological examination indicate that, as redefined here, Collabieae (including *Risleya*) is characterized by *Calanthe*-type velamen (hairy velamen), plants with rhizome, labellum usually saccate or spurred at base (except in *Eriodes* and *Risleya*), waxy pollinia, and elongate, flap-like rostellum.

### Phylogenetic relationships within Collabieae

It is unexpected that *Eriodes* is sister to the remaining genera in Collabieae (BP = 77, PP = 1.00). *Eriodes* has been neglected by most authors since it was established (such as [23], [34]), but can be easily distinguished from the remaining Collabieae by the combination of an epiphytic habit, distinct conical to globose pseudobulbs with 1–3 non-sheathing leaves, a lip without a spur, a column with a distinct foot, and eight pollinia. Most genera in Collabieae are terrestrial. Its distinctive morphology supports *Eriodes* as a sister of the remaining genera in Collabieae.

*Acanthephippium* is usually considered to be a member of Phajinae. Our results indicate that *Acanthephippium* is sister to other subclades of Collabiinae. This result remains to be tested further. *Tainia* is subdivided into two groups: one group, traditionally known as *Ania* (syn. *Ascotainia*), is sister to a clade formed by *Hancockia* (subclade II) and subclade I, whereas the other group is mixed with *Nephelaphyllum* and *Mischobulbum* in subclade I. These results agree well with their vegetative characters, such as shape and growing pattern of pseudobulbs, and vernation of the leaves. The group formed by *Nephelaphyllum*, *Mischobulbum* and *Tainia* spp. is strongly supported and is well characterized by morphological characters, such as ascending pseudobulbs articulated at their junction with either the petiole or scape, inflorescences arising on specialized leafless shoots, one convolute, petiolate, not sheathing leaf per pseudobulb, lip more or less concave at base, 8 pollinia, and no viscidium. *Ania* is characterized by conical to ovoid pseudobulbs often growing above ground, plicate leaf with long petiole and sheathing at base, spurred lip, and 8 pollinia without a viscidium. The monotypic *Hancockia* is characterized by a 1-flowered inflorescence arising from the top of the pseudobulb, pedicel 2.5 cm long, and pollinia with stipe and viscidium. Based on these findings, we support the separation of *Tainia* in two genera: *Tainia* (including *Nephelaphyllum* and *Mischobulbum*) and *Ania*.

Phajinae is subdivided into 4 subclades. *Gastrorchis* is sister to subclade V, which is supported by morphological characters, such as the few-leaved, pseudobulbous stem, pleated leaves, and eight waxy pollinia. *Calanthe* is subdivided into three groups, one consisting of sect. *Calanthe* together with sect. *Ghiesbreghtia*, the second consisting of sect. *Styloglossum*, and the third of subgenus *Preptanthe*. Section *Ghiesbreghtia* nests within section *Calanthe* in subclade V (Figure 2, 3), which is supported by gross morphological characters, such as their short stem-like pseudobulbs, persistent bracts, inflorescence more or less pubescent and flowers, and lip usually spurred. *Calanthe delavayi* has been considered as an intermediate between *Phaius* and *Calanthe* on column structure and sometimes transferred to *Phaius* [35], but our results indicate that it belongs to *Calanthe* sect. *Calanthe* (Figure 2, 3). *Cephalantheropsis* is nested within *Calanthe* sect. *Styloglossum* in subclade VI, which is supported by several morphological characters, such as an elongate rhizome, leaf sheaths forming a distinct pseudostem, and inflorescence arising from the base of the indistinct pseudobulb (Figure 2, S2).

Based on the morphological and molecular evidence, our results indicate that *Calanthe* is paraphyletic with respect to *Cephalantheropsis*, *Phaius* and *Gastrorchis*. Morphologically, *Calanthe* differs from *Phaius* in having the column more or less united to its apex with the lip, while *Phaius* is characterized by having the apex of the column free from the lip. However, our results indicated that adnation of the lip to the column evolved several times independently. Some species, including *Calanthe delavayi*, have an intermediate column type between these two states. There are two alternative approaches for the circumscription of *Calanthe* and its infrageneric groups. The first option is to consider each subclade in clade II as distinct genera, and narrow *Calanthe* to include only *Calanthe* sect. *Calanthe* and sect. *Ghiesbreghtia* in subclade V. In this approach, at least four genera, *Calanthe*, *Cephalantheropsis*, *Gastrorchis*, together with a new genus to include the former subgenus *Preptanthe*, should be recognized. The second option is to define *Calanthe* in a broad sense: for *Calanthe* s.l. to remain as monophyletic, then the genera *Cephalantheropsis*, *Gastrorchis*, and *Phaius* should have to be included in it.

Based on morphological and molecular systematics evidence, and in the interest of nomenclatural stability, we prefer to circumscribe *Calanthe* in the broad sense, including *Calanthe s.s.*, *Cephalantheropsis*, *Gastrorchis*, and *Phaius*.

### Evolution from an epiphytic to a terrestrial habit

A shift from the epiphytic to the terrestrial habit has evolved many times in Orchidaceae [4]. Collabieae is nested within a superclade consisting of more than 15 000 species (see [6]), of which 95% are epiphytic. However, Collabieae are predominantly terrestrial orchids, and even an alpine mycoheterotrophic genus has evolved, while the epiphytic *Eriodes* is sister to remaining terrestrial genera. Our results of morphological characters reconstruction indicated that terrestrial Collabieae have evolved from epiphytic ancestors (Figure 2, S2). This evolution in habit has been accompanied by a variety of pseudobulb shapes, ranging from heteroblastic, petiole-like in *Tainia* and *Hancockia* to homoblastic, fleshy and swollen in certain species of *Phaius* (e.g. *P. takeoi*), while pseudobulbs are even absent in some taxa. This great diversity in vegetative morphology and complicated evolutionary patterns of morphological characters have contributed to the confusion surrounding the systematics of the tribe Collabieae, which only now is becoming better understood.

## Materials and Methods

### Ethics statement

The species collected here are not included in the checklist of Chinese Protected Species. The fieldwork was conducted under the permission of the authority of each natural reserve, specifically Gaoligongshan National Nature Reserve (Yunnan, China), Xishuanbanna National Natural Reserve (Yunnan, China), Huanglianshan National Nature Reserve (Yunnan, China) and Wuzhishan National Nature Reserve (Hainan, China). No specific permits were required for the described field studies.

### Taxon and gene sampling

For the subfamily-wide analysis, a total of 96 genera (Table S1 in File S1) were sampled, representing all tribes of subfamily Epidendroideae. In total, 128 accessions of Epidendroideae taxa were analyzed, including two accessions each of *Phaius flavus*, *Risleya atropurpurea*, and *Tainia hookeriana*. Outgroups include 4 species from subfamily Orchidoideae and 1 species from Cypripedioideae. We sequenced 4674 bp of chloroplast DNA, including the *rbcL* and *psaB* genes, and the *matK* pseudogene. All terminal taxa represent single species and include at least two of the three DNA markers. Voucher information and GenBank accession numbers are listed in Table S1 (in File S1).

A second series of analyses focused on the tribe Collabieae (see Table S2 in File S1). We sampled 14 out of 18 genera of Collabieae as circumscribed in Pridgeon et al. [19]. The reduced matrix included 49 ingroup species and 3 outgroups. We analyzed 6775 bp of chloroplast DNA, including the *rbcL* and *psaB* genes, the *matK* pseudogene, and the *trnH*-*psbA* region. Voucher information and GenBank accession numbers are listed in Table S2 (in File S1).

The primers used in both series of analyses are listed in Table S3 (in File S1).

### Phylogenetic analysis

Sequences were aligned using the default parameters in Clustal X v1.83 [36] and manually adjusted with BioEdit v5.0.9 [37]. Phylogenetic analyses of the combined dataset were carried out using parsimony (PAUP* v4.0b10) [38], and Bayesian inference (BI; MrBayes v3.2.0) [39]. Parsimony heuristic searches were performed with 1000 random sequence addition replicates, tree-bisection-reconnection (TBR) branch swapping, MulTrees in effect, and steepest descent off, saving all minimum length trees (MULPARS on). Internal branch support under MP was estimated by using 1000 bootstrap (BS) replicates; the starting trees were obtained by random addition with ten replicates for each replication, TBR branch swapping, and MULPARS in effect.

For BI analyses, we partitioned our data a priori on the basis of gene identity and, for coding regions, codon position (Table S4 in File S1). Based on Bayes factors, the partitioning strategy (*rbcL*, *matK*, *psaB*, and *trnH-psbA*) was identified as optimal for our data and was applied in all subsequent Bayesian analyses. Initial analyses providing data for comparison of the different partition strategies were run for 3 000 000 generations, and analyses applying the final best-fit model were run for 5 000 000 generations. Runs were started from a random tree sampled every 1000 generations of the MCMC chain, with default priors and the option prset/ratepr set as variable. Each parameter estimation obtained from the results of two runs was checked in Tracer v1.5 (http://tree.bio.ed.ac.uk/software/tracer) to ascertain whether they had obtained proper effective sample size and to verify that stationary state had been reached. Trees from the first 10% of generations were discarded as burn-in. The remaining trees were combined to build a 50% majority-rule consensus tree. Bayesian inference was run on CIPRES [40]. The data matrix and phylogenetic trees have been submitted to TreeBase (http://purl.org/phylo/treebase/phylows/study/TB2:S14958).

### Morphological data analysis

A total of 41 characters were included in the analysis (Table S5 in File S1). Characters were coded for 40 species representing most genera in Collabieae. Nine characters were constant and 27 were parsimony informative. A parsimony and Bayesian analyses with all characters equally weighted was conducted in PAUP* v4.0b10 [38] and MrBayes v3.2.0 [39]. The evolution of morphological characters was reconstructed using a maximum parsimony approach implemented in Mesquite v2.74 (http://mesquiteproject.org/mesquite/mesquite.html).

## Supporting Information

Figure S1 **Bayesian inference (left) and maximum parsimony (right) phylogenetic relationships of the tribe Collabieae based on morphological data.** Numbers at the nodes are posterior probabilities and bootstrap percentages (>50%), respectively.
(TIF)

Figure S2 **Reconstruction of morphological character among the tribe Collabieae.** The species orders are same to Figure 3.
(TIF)

File S1 **Tables.** Table S1. Taxa, voucher and GenBank accession numbers of Epidendroideae used in this study; Table S2. Taxa, voucher and GenBank accession numbers of Collabieae used in this study; Table S3. Primers used for amplification and sequencing in this study; Table S4. Partition analysis of Bayesian inference; Table S5. Morphological data matrix for the phylogenetic analysis.
(DOCX)
