# Correction: Phylogenetics of Tribe Collabieae (Orchidaceae, Epidendroideae) Based on Four Chloroplast Genes with Morphological Appraisal

**DOI:** 10.1371/journal.pone.0098721

**Published:** 2014-05-23

**Authors:** The PLOS ONE Staff

There is an error in some of the references listed for this article. The correct references are provided by the authors below:

1. Garay L (1960) On the origin of the Orchidaceae. Botanical Museum Leaflets 19: 57-88.

16. Xiang X-G, Li D-Z, Jin X-H, Hu H, Zhou H-L, et al. (2012) Monophyly or Paraphyly — The Taxonomy of Holcoglossum (Aeridinae: Orchidaceae). PloS One 7: 10.1371/journal.pone.0052050.

18. Chen SC, Tsi ZH (1999) Flora Republicae Popularis Sinicae. Vol. 18. Beijing: Science Press. pp.229-333.

19. Pridgeon AM, Cribb PJ, Chase MW, Rasmussen FN (2005) Genera Orchidacearum Vol. 4. Epidendroideae (Part 1). Oxford: Oxford University Press. Pp.116-161.

21. Chen SC, Liu ZJ, Zhu GH, Lang KY, Tsi ZH, et al. (2009) Flora of China. Vol. 25. Beijing: Science Press. pp. 280-314.

27. Smith JJ (1912) Die Gruppe der Collabiinae. Bulletin du Jardin Botanique de Buitenzorg, Ser II 8. 5-6.

29. Seidenfaden G (1986) Orchid genera in Thailand XIII. Thirty-three epidendroid genera. Opera Botanica 89: 1-214.

31. Gagnepain F (1932) Orchidacées nouvelles ou critiques I—Tainia ou Nephelaphyllum? Bulletin du Muséum d'Histoire Naturelle Paris, sér 2: 705-710.

32. Dressler R (1993) Phylogeny and classification of the orchid family. Portland, OR.: Dioscorides Press.

34. Schlechter R (1919) Orchdeologiae Sino-Japonicae Prodromus. Repert Sp Nov Reg Veg 4: 178-182.
