# Supplementary figures and images for: Phylogenetics of Tribe Collabieae (Orchidaceae, Epidendroideae) Based on Four Chloroplast Genes with Morphological Appraisal
Source: PLoS One. 2014 Jan 31;9(1):e87625. doi: 10.1371/journal.pone.0087625 (PMC3909211; doi:10.1371/journal.pone.0087625)

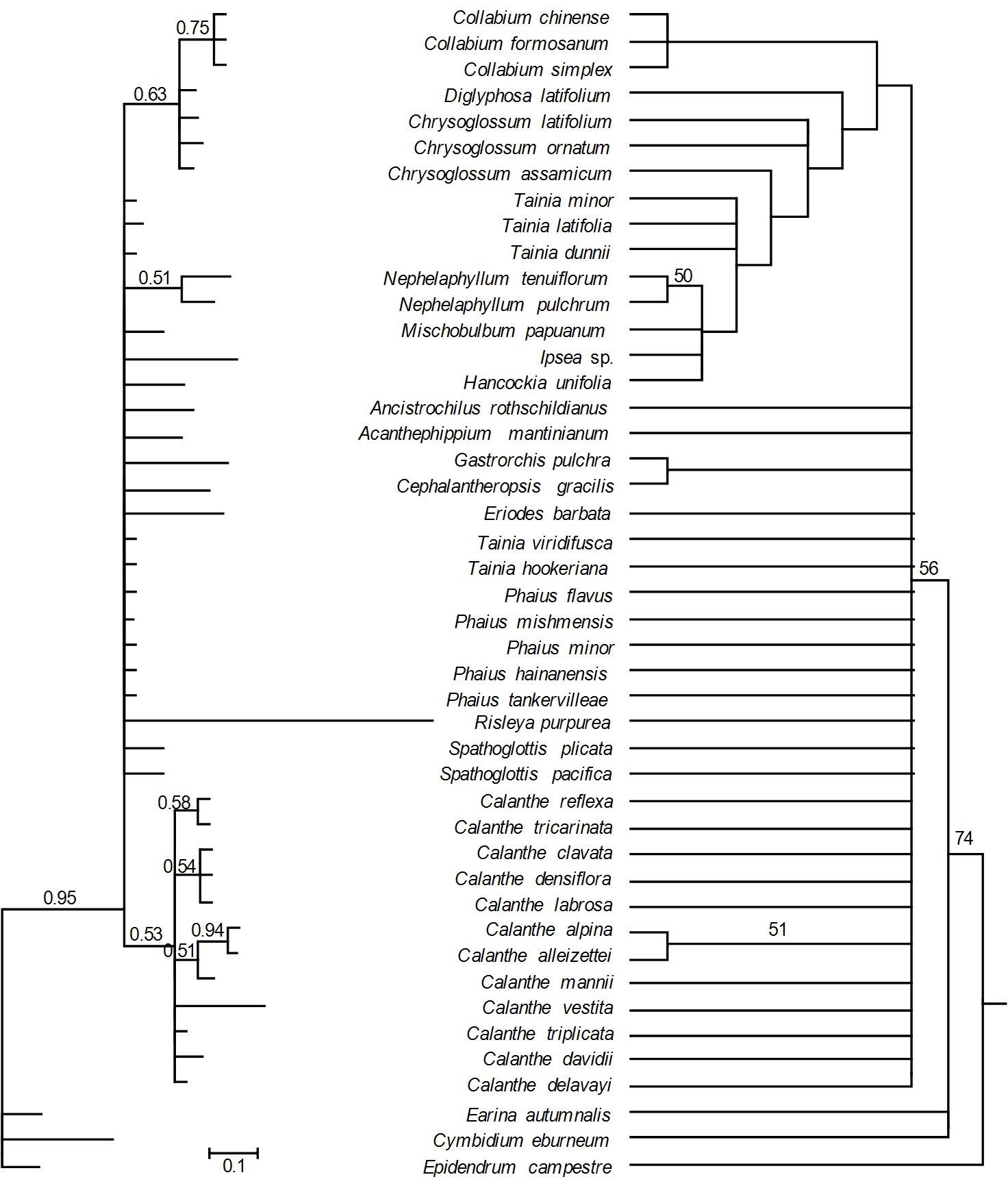

Supplement: Figure S1 — Bayesian inference (left) and maximum parsimony (right) phylogenetic relationships of the tribe Collabieae based on morphological data. Numbers at the nodes are posterior probabilities and bootstrap percentages (>50%), respectively. (TIF) [file pone.0087625.s001.tif]

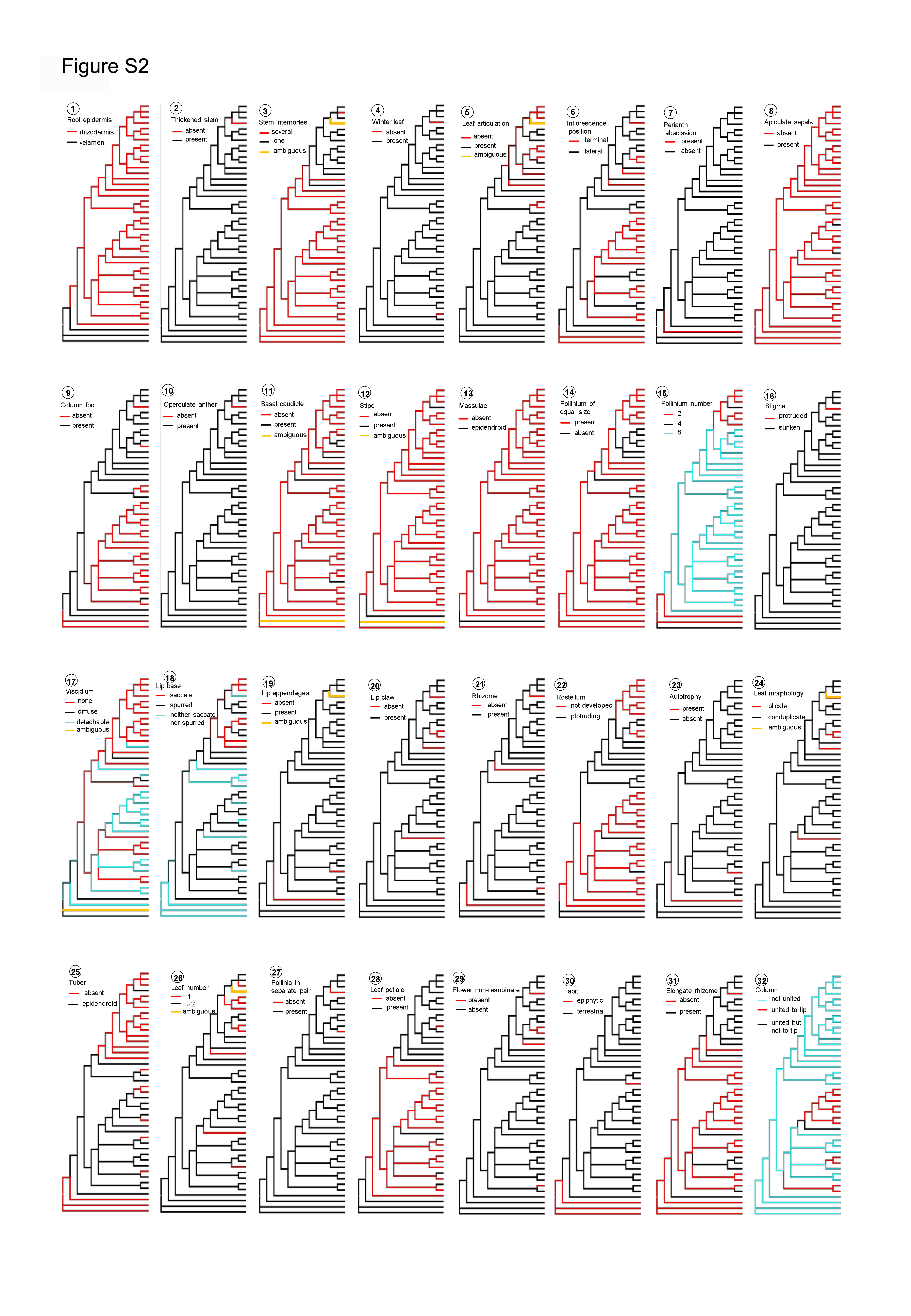

Supplement: Figure S2 — Reconstruction of morphological character among the tribe Collabieae. The species orders are same to Figure 3. (TIF) [file pone.0087625.s002.tif]
