# Supplementary material for: Phylogenetics of Tribe Collabieae (Orchidaceae, Epidendroideae) Based on Four Chloroplast Genes with Morphological Appraisal
Source: PLoS One. 2014 Jan 31;9(1):e87625. doi: 10.1371/journal.pone.0087625 (PMC3909211; doi:10.1371/journal.pone.0087625)
Supplement: File S1 — Tables. Table S1. Taxa, voucher and GenBank accession numbers of Epidendroideae used in this study; Table S2. Taxa, voucher and GenBank accession numbers of Collabieae used in this study; Table S3. Primers used for amplification and sequencing in this study; Table S4. Partition analysis of Bayesian inference; Table S5. Morphological data matrix for the phylogenetic analysis. (DOCX) [file pone.0087625.s003.docx]

**Table S1. Taxa analyzed in the subfamily-wide analysis, voucher and GenBank accession numbers.**

| Species | Voucher_*rbcL*/*matK*/*psaB* | *rbcL* | *matK* | *psaB* |
| --- | --- | --- | --- | --- |
| *Acanthephippium mantinianum* Linden & Cogn. | Chase O-397 (K) | AF074100^1^ | AF263618^2^ | AY380927^3^ |
| *Aeranthes* sp. | Chase O-880 (K)/Chase O-154 (K)/FLAS-living s.n. | AF074104^1^ | AY368390^4^ | AY380932^3^ |
| *Ancistrochilus rothschildianus* J.O'Brien | Chase O-669 (K) | AF264152^2^ | AF263620^2^ | - |
| *Angraecum* sp. | Jarrell s.n. (unknown)/Jarrell s.n. (unknown)/Cameron 2129 (NY) | AF074106^1^ | AF263621^2^ | AY380933^3^ |
| *Anthogonium gracile* Lindl. | Chase O-538 (K) | AF264153^2^ | AF263622^2^ | - |
| *Arethusa bulbosa* L. | Goldman 446 (TEX) | AF264154^2^ | AF263624^2^ | AY380938^3^ |
| *Arpophyllum giganteum* Hartw. ex Lindley | Chase O-586 (K)/Chase s.n. (K)/ Chase O-586 (K) | AF074110^1^ | AF265485^5^ | AY380939^3^ |
| *Arundina graminifolia* (D.Don) Hochr. | Chase O-395 (K)/Szlachetko s.n (unknown)/-. | AF074111^1^ | EF079333^6^ | - |
| *Bletia catenulata* Ruiz & Pavon | Forest 10 (ESA)/Forest 10 (ESA)/ Chase O-581 (K) | AF518024^7^ | AY121720^8^ | AY380942^3^ |
| *Bletilla striata* Rchb.f. | Chase O-556 (K)/Szlachetko s.n. (unknown)/ Chase O-556 (K) | AF074114^1^ | EF079331^6^ | AY380943^3^ |
| *Bracisepalum selebicum* J.J.Sm. | -/Leiden cult. 20446 (L)/- | - | AY003873^9^ | - |
| *Bulbophyllum lobbii* Lindl. | Chase O-474 (K) | AF074115^1^ | AY121740^8^ | AY380944^3^ |
| *Cadetia taylori* (F.Muell.) Schltr. | Shiraishi 132 (unknown)/Heidelberg BG 120066 (unknown)/NYBG-living 191-75a | D58406^10^ | EF079346^6^ | AY380945^3^ |
| *Calanthe tricarinata* Lindl. | Chase O-820 (K) | AF264160^2^ | AF263633^2^ | - |
| *Calopogon tuberosus* (L.) Britton, Sterns & Poggenb. | Goldman 532 (BH, TEX) | AF264161^2^ | AF263635^2^ | - |
| *Calypso bulbosa* (L.) Oakes | Chase O-490 (K)/ Chase O-490 (K)/Grant 3763 (NEU) | AF264162^2^ | EF525689^11^ | AY380950^3^ |
| *Catasetum expansum* Rchb. f. | Chase O-224 (K)/F0859 (FLAS)/NYBG-living 3199 | AF074121^1^ | AF263637^2^ | AY380951^3^ |
| *Cattleya* sp. | Chase O-282 (K)/ Chase O-282 (K)/NYBG-living 3052 | AF074122^1^ | AY263638^2^ | AY380952^3^ |
| *Cephalanthera damasonium* (Miller) Druce | Chase O-575 (K) | AF074123^1^ | AY368396^4^ | - |
| *Cephalanthera nanchuanica* (S.C.Chen) X.H.Jin & X.G.Xiang | Jin XH 9383 (PE) | JN706686^12^ | JN706689^12^ | - |
| *Cephalantheropsis gracilis* (Lindl.) S. Y. Hu | Jin XH 057 (PE) | KF852714 | KF852670 | KF850602 |
| *Chelonistele amplissima* (Ames & C. Schweinf.) Carr | -/Leiden cult. 26834 (L)/- | - | AF302695^13^ | - |
| *Chelonistele sulphurea* (Blume) Pfitzer | -/Leiden cult. 21528 (L)/- | - | AF302694^13^ | - |
| *Chrysoglossum assamicum* Hook. f. | Jin XH 9320 (PE) | KF852715 | KF852671 | KF852603 |
| *Chrysoglossum ornatum* Blume | Jin XH 11874 (PE) | KF852732 | KF852687 | KF852620 |
| *Chrysoglossum latifolium* (Blume) Benth. | Kurzweil H. & Lwin S. 2555 (SING) | KF852733 | KF852688 | KF852621 |
| *Chysis bractescens* Lindl. | Chase O-436 (K)/Wien BG (unknown)/NYBG-living 1129 | AF074126^1^ | EF079351^6^ | AF380956^3^ |
| *Coelia macrostachya* Lindl. | Chase O-817 (K) | AF518055^7^ | AY121743^8^ | - |
| *Coelogyne cristata* Lindl. | Chase O-491 (K)/ Chase O-491 (K)/NYBG-living 3025 | AF074133^1^ | AF263644^2^ | AY380967^3^ |
| *Coelogyne asperata* Lindl. | -/Leiden cult. 22279 (L)/- | - | AY003881^9^ | - |
| *Collabium chinensis* (Rolfe)T. Tang et F.T. Wang | ST-ET 2373 (PE) | KF852716 | KF852672 | KF852604 |
| *Collabium formosanum* Hayata | YN-ET 310 (PE) | KF852717 | KF852673 | KF852605 |
| *Collabium* sp. | Chase O-821 (K) | AF264163^2^ | AF263645^2^ | - |
| *Collabium simplex* Rchb. f. | -/Kocyan 991017-1-04 (L)/- | - | AY557200^14^ | - |
| *Comparettia falcata* Poepp. & Endl. | Whitten 2688 (FLAS) | FJ534237^15^ | FJ565090^16^ | FJ534359^15^ |
| *Cymbidium* sp. | Cameron 1098 (NY)/ Chase O-1505 (K)/ Chase O-1098 (K) | AY368356^4^ | AF470470^17^ | AY380978^3^ |
| *Dendrobium fimbriatum* Hook. | Jin XH s.s.14 (PE) | KF177603 | KF143671 | - |
| *Dendrochilum* sp. | Chase O-624 (K) | AF264164^2^ | AY121730^8^ | - |
| *Diaphananthe rutila* (Rchb. f.) Summerh. | Jarrell s.n. (unknown)/Jarrell s.n. (unknown)/NYBG-living 543 | FJ074147^1^ | AY368403^4^ | AY380985^3^ |
| *Dilochia* sp. | Chase O-672 (K) | AF264165^2^ | AF263653^2^ | - |
| *Dilomilis montana* (Sw.) Summerh. | Chase O-206 (K)/unknown/ Chase O-206 (K) | AF074150^1^ | AF263765^2^ | AY380987^3^ |
| *Eleorchis japonica* (A.Gray) Maek | Goldman 1103 (TEX) | AF264166^2^ | AF263657^2^ | - |
| *Elleanthus* sp. | Chase O-374 (K) Chase O-374 (K)/NYBG-living 1437 | AF074156^1^ | AF263658^2^ | AY380994^3^ |
| *Entomophobia kinabaluensis* (Ames) de Vogel | Leiden 970404 (L) | AF518036^7^ | AY121733^8^ | - |
| *Epidendrum campestre* Lindl. | Borba EL 553 (UEC)/unknown/NYBG-living 2017 | AF518060^7^ | AF263781^32^ | AY380996^3^ |
| *Epipactis* sp. | unknown/ Chase O-199 (K)/Cameron 1097 (NY) | FJ454877^19^ | AF263659^2^ | AY380998^3^ |
| *Eria ferruginea* Teijsm. & Binn. | Chase O-590 (K)/ Chase O-590 (K)/NYBG-living 28106 | AF074164^1^ | AF263660^2^ | AY381004^3^ |
| *Eriodes barbata* (Lindley) Rolfe | Kurzweil H. & Lwin S. 2542 (SING) | KF852718 | KF852674 | KF852606 |
| *Fernandezia ionanthera* (Rchb.f. & Warsz.) Schltr. | Whitten 1701 (FLAS) | FJ534219^15^ | FJ565010^16^ | FJ534341^15^ |
| *Galeandra devoniana* R.H. Schomb. ex Lindl. | Chase O-382 (K) | AF074171^1^ | AY368408^4^ | AY381011^3^ |
| *Gastrorchis pulchra* Humbert & H.Perrier | -/Heidelberg BG 104634 (unknown)/- | - | EF079305^6^ | - |
| *Geesinkorchis phaiostele* (Ridl.) de Vogel | -/Leiden cult. 30700 (L)/- | - | AF302698^13^ | - |
| *Glomera pulchra* (Schltr.) J.J.Sm. | -/Leiden 960835 (L)/- | - | AF518053^7^ | - |
| *Glomera* sp. | Chase O-555 (NCU)/ Chase O-555 (K)/- | AF074172^1^ | AY121742^8^ | - |
| *Glomera* sp. | SH-2010 (unknown)/-/Motley T 2200 (NY) | AB586467^20^ | - | AY381013^3^ |
| *Grandiphyllum auriculum* (Vell.) Docha Neto | Whitten 3567 (FLAS) | FJ534200^15^ | FJ565155^16^ | FJ534322^15^ |
| *Hancockia unifolia* Rolfe | YN-ET 1006 (PE) | KF852719 | KF852675 | KF852607 |
| *Helcia brevis* (Rolfe) Dodson | Chase 86160 (K)/Hannover BG (unknown)/ Chase 86160 (K) | FJ534148^15^ | EF079229^6^ | FJ534336^15^ |
| *Ionopsis minutiflora* (Dodson & N. Williams) Pupulin | Whitten 3430 (FLAS) | FJ534024^15^ | FJ565047^16^ | FJ534285^15^ |
| *Kegeliella kupperi* Mansfeld | Chase O-495 (K)/Heidelberg BG 122428 (unknown)/ Chase O-495 (K) | AF074181^1^ | EF079232^6^ | FJ534326^15^ |
| *Liparis bootanensis* Griff. | Jin XH 11013 (PE) | KF852720 | KF852676 | KF852608 |
| *Liparis cespitosa* (Thou.) Lindl. | Jin XH 11556 (PE) | KF852721 | KF852677 | KF852609 |
| *Liparis distans* C.B. Clarke | Jin XH 9097 (PE) | KF852722 | KF852678 | KF852610 |
| *Liparis lilifolia* (Bl.) Lindl. | Chase O-214 (K)/McCartney s.n. (unknown)/- | AF074183^1^ | AY907156^21^ | - |
| *Listera smallii* Wiegand | Cameron 1001 (NCU)/Cameron 1001 (NCU)/Paris-living s.n. | AF074184^1^ | AF521058^7^ | AY381027^3^ |
| *Lycaste cruenta* Lindl. | unknown/Whitten 97021 (FLAS)/NYBG-living 79449 | AF074185^1^ | AF239438^22^ | AY381028^3^ |
| *Lycomormium squalidum* (Poepp. & Endl.) Rchb. f. | Chase O-273 (K)/ Chase O-273 (K)/FLAS-living 87056 | AF074186^1^ | AY368414^4^ | AY381029^3^ |
| *Macradenia rubescens* Barb.Rodr. | Gerlach 98-2069 (M) | FJ534181^15^ | FJ564839^16^ | FJ534303^15^ |
| *Malaxis cylindrostachya* Lindl. | SBB-0907 (unknown)/SBB-0906 (unknown)/- | JN005437^18^ | JN004421^18^ | - |
| *Malaxis latifolia* J.E. Smith | Jin XH 7750 (PE) | KF852723 | KF852679 | KF852611 |
| *Malaxis monophyllos* (L.) Sw. | Jin XH 13156 (PE) | KF852724 | KF852680 | KF852612 |
| *Malaxis spicata* Sw. | Chase O-377 (K)/ Chase O-377 (K)/McCartney s.n. (unknown) | AF074188^1^ | AY368415^4^ | AY381031^3^ |
| *Masdevallia uniflora* Ruiz & Pavon | Kew 1997-5356 (K)/ Kew 1997-5356(K)/NYBG-living 5377 | AF518040^7^ | AF265446 | AY381032^3^ |
| *Meiracyllium trinasutum* Rchb. f; | Chase O-202 (K)/Heidelberg BG 120031 (unknown)/NYBG-living 5178 | AF074192^1^ | EF079317^6^ | AY381037^3^ |
| *Miltonia regnellii* Rchb. f. | Chase 86059 (K)/Whitten 92014 (FLAS)/ Chase 86059 (K) | FJ534193^15^ | AF239491^22^ | FJ534315^15^ |
| *Mischobulbum papuanum* (J.J. Sm.) Schltr. | Reeve 1150 (K) | AF264169^2^ | AF263672^2^ | - |
| *Nabaluia angustifolia* de Vogel | -/Leiden cult. 26217 (L)/- | - | AF302699^13^ | - |
| *Neofinetia falcata* (Thunb. ex A. Murray) H. H. Hu | Jarrell 3 (unknown)/Kocyan 010824 (Z)/NYBG-living 5097 | AF074197^1^ | EF655782^33^ | AY381041^3^ |
| *Neogyne gardneriana* (Lindl.) Rchb. f. | -/Leiden cult. 970729 (L)/- | - | AF302700^13^ | - |
| *Nephelaphyllum pulchrum* Blume | Chase O-668 (K) | AF518049^7^ | AF263674^2^ | - |
| *Nephelaphyllum tenuiflorum* Blume | Jin XH 058 (PE) | KF852734 | KF852689 | KF852622 |
| *Nervilia* sp. | Chase O-580 (K)/Chase 9057 (K)/ Chase O-580 (K) | AF074199^1^ | AY368420^4^ | AY381044^3^ |
| *Nohawilliamsia orthostates* (Ridl.) M.W.Chase & Whitten | Chase 8175 (K) | FJ534189^15^ | FJ563950^16^ | FJ534311^15^ |
| *Oberonia caulescens* Lindl. | Jin XH 13340 (PE) | KF852725 | KF852681 | KF852613 |
| *Oberonia ensiformis* (Sm.) Lindl. | Jin XH 11022 (PE) | KF852726 | KF852682 | KF852614 |
| *Oliveriana brevilabia* (C. Schweinf.) Dressler & N. H. Williams | Whitten 1737 (FLAS)/Heidelberg BG 123415 (unknown)/Whitten 1737 (FLAS) | FJ534174^15^ | EF079202^6^ | FJ534296^15^ |
| *Oncidium harryanum* (Rchb.f.) M.W. Chase & N.H. Williams | Chase 86165 (K)/Eciagenera (unknown)/ Chase 86165 (K) | FJ534144^15^ | EF079216^6^ | FJ534352^15^ |
| *Otochilus albus* Lindl. | -/unknown/- | - | HQ130493^23^ | - |
| *Otochilus* sp. | -/unknown/- | - | HQ130494^23^ | - |
| *Palmorchis trilobulata* L.O. Williams | Chase O-462 (K)/Dressler s.n. (FLAS)/ Chase O-462 (K) | AF074206^1^ | AJ310052^24^ | AY381051^3^ |
| *Panisea* *tricallosa* Rolfe | -/Leiden cult. 970828 (L)/- | - | AF302701^13^ | - |
| *Phaius flavus* (Bl.) Lindl. | Jin XH 10359 (PE) | KF852727 | KF852683 | KF852615 |
| *Phaius flavus* (Bl.) Lindl. | Chase O-325 (K) | AF074210^1^ | AF263676^2^ | - |
| *Phaius tancarvilleae* (Banks ex L'Her.) Blume | Jin XH 10370 (PE) | XXXX | XXXX | XXXX |
| *Pholidota imbricata* Lindl. | -/Leiden cult. 21540 (L)/- | - | AF302703^13^ | - |
| *Phreatia* sp. | Chase O-203 (K)/ Chase O-561 (K)/Cameron 2048 (NY) | AF074214^1^ | AY368425^4^ | AY381056^3^ |
| *Phymatidium falcifolium* Lindl. | Whitten 1848 (FLAS) | FJ534183^15^ | FJ563942^16^ | FJ534305^15^ |
| *Pleione chunii* C.L. Tso | van der Berg C290 (K) | AF518035^7^ | AY121732^8^ | - |
| *Pleione formosana* Hayata | Chase O-670 (K) | AF264173^2^ | AF263679^2^ | - |
| *Pleurothallis* sp. | Chase O-306 (K)/Hermans 2950 (K)/Cameron 1128 (NY) | AF074217^1^ | AF265456^25^ | AY381059^3^ |
| *Podochilus* sp. | Chase O-559 (K) | AF074218^1^ | AY121738^8^ | AY381060^3^ |
| *Polystachya pubescens* (Lindl.) Rchb. f. | Chase O-152 (K)/ Chase O-152 (K)/NYBG-living 4470-95a | AF074222^1^ | AY368426^4^ | AY381064^3^ |
| *Prosthechea abbreviata* (Schltr.) W.E. Higgins | Brieger Coll. 10092 (ESA)/unknown/- | AF518063^7^ | AF263757^32^ | - |
| *Psychopsis sanderae* (Rolfe) Lückel & Braem | Chase 96126 (K) | FJ534141^15^ | FJ564712^16^ | FJ534264^15^ |
| *Risleya atropurpurea* King et Pantl. | DLJ-ET 1675 (PE) | KF852728 | KF852684 | KF852616 |
| *Risleya atropurpurea* King et Pantl. | Jin XH 9174 (PE) | KF852729 | KF852685 | KF852617 |
| *Rodriguezia batemanii* Lindl. | Whitten 1615 (FLAS) | FJ534211^15^ | FJ564975^16^ | FJ534333^15^ |
| *Rudolfiella* sp. | Whitten 1618 (FLAS) | FJ534212^15^ | FJ564977^16^ | FJ534334^15^ |
| *Sobralia macrantha* Lindl. | Chase O-200 (K)/ Chase O-200 (K)/NYBG-living 3557 | AF074228^1^ | AF263681^2^ | AY381076^3^ |
| *Solenidium portillae* Dalström & Whitten | Whitten 1812 (SEL) | FJ534206^15^ | FJ564956^16^ | FJ534328^15^ |
| *Spathoglottis pacifica* Rchb.f. | Motley 2277 (NY)/-/Motley 2277 (NY) | AY381134^3^ | - | AY381077^3^ |
| *Spathoglottis plicata* Blume | Motley 2277 (NY)/ Chase O-671 (K)/Motley 2277 (NY) | AY381134^3^ | AY368429^4^ | AY381077^3^ |
| *Tainia hookeriana* King & Pantl. | Jin XH 9285 (PE) | KF852756 | KF852711 | KF852668 |
| *Tainia hookeriana* King & Pantl. | Chase O-649 (K)/Heidelberg BG 125230 (unknown)/- | AF264176^2^ | EF079342^6^ | - |
| *Tainia minor* Hook. F. | Jin XH 10374 (PE) | KF852730 | KF852686 | KF852618 |
| *Thaia saprophytica* Seidenf. | Jin XH 10463 (PE) | JN706687^12^ | JN706690^12^ | - |
| *Thecostele alata* (Roxb.) E.C.Parish & Rchb. f. | Chase O-373 (K) | AY368371^4^ | AY368431^4^ | - |
| *Thunia alba* (Lindl.) Reichb. f. | Chase O-589 (K) | AF074233^1^ | AY121731^8^ | - |
| *Tipularia discolor* (Pursh) Nutt. | Freudenstein s.n. (unknown)/Doyle s.n. (unknown)/- | AF074234^1^ | AF263685^2^ | - |
| *Trichotosia ferox* Blume | Chase O-396 (K) | AF074235^1^ | AY368432^4^ | AY381085^3^ |
| *Tropidia* sp. | Chase O-211 (K)/ Chase O-211 (K)/Motley 2234 (NY) | AF074237^1^ | AF263686^2^ | AY381087^3^ |
| *Xerorchis amazonica* Schltr. | Romero 3014 (AMES) | AF074244^1^ | AF263688^2^ | AY381096^3^ |
| **Outgroups** |  |  |  |  |
| *Disa tripetaloides* (L.f.) N.E. Br. | Cameron 1047 (NCU)/Bytebier 2460 (K)/Cameron 2143 (NY) | AF074151^1^ | DQ415011^26^ | AY380988^3^ |
| *Diuris sulphurea* R.Br. | Chase O-554 (K) | AF074152^1^ | AF263655^2^ | AY380990^3^ |
| *Orchis quadripunctata* Cirillo ex Ten. | Chase O-911 (K) | AF074203^1^ | AY368385^4^ | AY381048^3^ |
| *Spiranthes spiralis* (L.) Chevall. | Bateman s.n. (K)/Bateman s.n. (unknown)/Alvarez-Molina 3015 (QCNE) | AJ542434^27^ | AJ543918^27^ | FJ571255^28^ |
| *Phragmipedium longifolium* (Warsc. & Rchb.f) Rolfe | Albert 18 (NCU)/Whitten 2802 (FLAS)/Cameron 1107 (NY) | AF074212^1^ | AY918831^29^ | AY381055^3^ |

Note: One voucher represent all sequences from the same specimen.

**Table S2. Taxa analyzed in the Collabieae analysis, voucher and GenBank accession numbers.**

| Species | Voucher_*rbcL*/*matK*/*psaB*/*trnH*-*psbA* | *rbcL* | *matK* | *psaB* | *trnH-psbA* |
| --- | --- | --- | --- | --- | --- |
| *Acanthephippium mantinianum* Linden & Cogn. | Chase O-397 (K) | AF074100^1^ | AF263618^2^ | - | - |
| *Ancistrochilus rothschildianus* O'Brien | Chase O-669 (K) | AF264152^2^ | AF263620^2^ | - | - |
| *Calanthe alleizettei* Gagnepain | Jin XH -S120 (PE) | KF852735 | KF852690 | KF852623 | KF852636 |
| *C. alpina* Hook. f. ex Lindl. | Jin XH 7042 (PE) | KF852736 | KF852691 | - | KF852637 |
| *C. argenteo-striata* C. Z. Tang & S. J. Cheng | Jin XH 13466 (PE) | KF852737 | KF852692 | KF852624 | KF852638 |
| *C. brevicornu* Lindl. | Jin XH 7012 (PE) | KF852738 | KF852693 | KF852625 | KF852639 |
| *C. calanthoides* (A.Rich. & Galeotti) Hamer & Garay | Chase O-819 (K) | AF264159^2^ | AF263632^2^ | - | - |
| *C. clavata* Lindl. | Kurzweil H. & Lwin S. 2554 (SING) | KF852739 | KF852694 | - | KF852640 |
| *C. davidii* Franch. | Jin XH 7039 (PE) | KF852740 | KF852695 | KF852626 | KF852641 |
| *C. delavayi* Finet | Jin XH, Jin WT & Xu SZ 13133 (PE) | KF852741 | KF852696 | KF852627 | KF852642 |
| *C. densiflora* Lindl. | Jin XH -S119 (PE) | KF852742 | KF852697 | - | KF852643 |
| *C. graciliflora* Hayata | Jin XH 9574 (PE) | KF852743 | KF852698 | KF852628 | KF852644 |
| *C. herbacea* Lindl. | YN-ET 731 (PE) | KF852744 | KF852699 | KF852629 | KF852645 |
| *C. labrosa* (Rchb.f.) Rchb. f. | Jin XH 9438 (PE) | KF852745 | KF852700 | KF852630 | KF852646 |
| *C. mannii* Hook. f. | Jin XH 20071228 (PE) | KF852746 | KF852701 | - | KF852647 |
| *C. reflexa* Maxim. | SET-ET 460 (PE) | KF852747 | KF852702 | KF852631 | KF852649 |
| *C. tricarinata* Lindl. | SET-ET 505 (PE) | KF852748 | KF852703 | - | KF852650 |
| *C. tricarinata* Lindl. | Chase O-820 (K) | AF264160^2^ | AF263633^2^ | - | - |
| *C. triplicata* (Willem.) Ames | YN-ET 1100 (PE) | KF852731 | KF852713 | - | KF852648 |
| *C. vestita* Lindl. | Chase O-207 (K) | AF074117^1^ | AF263634^2^ | - | - |
| *Cephalantheropsis gracilis* (Lindl.) S. Y. Hu | Jin XH 057 (PE) | KF852714 | KF852670 | KF852602 | KF852651 |
| *Chrysoglossum assamicum* (Hook.f.) Seidenf*.* | Jin XH9320 (PE) | KF852715 | KF852671 | KF852603 | KF852654 |
| *C. latifolium* (Blume) Benth. & Hook.f. | Kurzweil H. & Lwin S. 2555 (SING) | KF852733 | KF852688 | KF852621 | KF852652 |
| *C. ornatum* Bl. | Jin XH 11874 (PE) | KF852732 | KF852687 | KF852620 | KF852653 |
| *Collabium chinense* (Rolfe) T. Tang & F. T. Wang | ST-ET 2373 (PE) | KF852716 | KF852672 | KF852604 | KF852655 |
| *C. formosanum* Hayata | YN-ET 310 (PE) | KF852717 | KF852673 | KF852605 | KF852656 |
| *C. simplex* Rchb.f. | -/Kocyan A. 991017-1-04 (L)/-/- | - | AY557200^14^ | - | - |
| *Eriodes barbata* (Lindley) Rolfe | Kurzweil H. & Lwin S. 2542 (SING) | KF852718 | KF852674 | KF852606 | KF852657 |
| *Gastrorchis pulchra* Humbert & H.Perrier | -/Heidelberg BG 104634 (unknown)/-/- | - | EF079305^6^ | - | - |
| *Hancockia unifolia* Rolfe. | YN-ET 1006 (PE) | KF852719 | KF852675 | KF852607 | KF852658 |
| *Mischobulbum papuanum* (J.J.Sm.) Schltr. | Reeve 1150 (K) | AF264169^2^ | AF263672^2^ | - | - |
| *Nephelaphyllum pulchrum* Blume | Chase O-668 (K) | AF518049^7^ | AF263674^2^ | - | - |
| *Nephelaphyllum tenuiflorum* Blume | Jin XH 058 (PE) | KF852734 | KF852689 | KF852622 | KF852659 |
| *Phaius flavus* (Bl.) Lindl. | Jin XH 10359 (PE) | KF852727 | KF852683 | KF852615 | KF852663 |
| *P. flavus* (Bl.) Lindl. | Chase O-325 (K) | AF074210^1^ | AF263676^2^ | - | - |
| *P. hainanensis* C. Z. Tang & S. J. Cheng | Jin XH 20091028 (PE) | KF852751 | KF852706 | - | KF852662 |
| *P. mishmensis* (Lindl. & Paxt.) Rchb.f. | Yu SX 548 (PE) | KF852750 | KF852705 | - | KF852661 |
| *P. tancarvilleae* (Banks ex L'Herit.) Bl. | Jin XH 060 (PE) | KF852749 | KF852704 | - | KF852660 |
| *P. tancarvilleae* (Banks ex L'Herit.) Bl. | Jin XH 10370 (PE) | KF852752 | KF852707 | KF852632 | KF852664 |
| *Risleya atropurpurea* King et Pantl. | DLJ-ET 1675 (PE) | KF852728 | KF852684 | KF852616 | - |
| *R. atropurpurea* King et Pantl. | Jin XH 9174 (PE) | KF852729 | KF852685 | KF852617 | - |
| *Spathoglottis pacifica* Rchb.f. | Motley 2277 (NY)/-/-/- | AY381134^3^ | - | - | - |
| *S. plicata* Blume | Motley 2277 (NY)/ Chase O-671 (K)/-/- | AY381134^3^ | AY368429^4^ | - | - |
| *Tainia dunnii* Rolfe | Jin XH 9761 (PE) | KF852753 | KF852708 | KF852633 | KF852665 |
| *T. hookeriana* King & Pantl. | Jin XH 9285 (PE) | KF852756 | KF852711 | KF852619 | KF852668 |
| *T. hookeriana* King & Pantl. | Chase O-349 (K)/Heidelberg BG 125230 (unknown)/-/- | AF264176^2^ | EF079342^6^ | - | - |
| *T. latifolia* (Lindl.) Rchb.f. | Jin XH 13671 (PE) | KF852754 | KF852709 | KF852634 | KF852666 |
| *T. minor* Hook. f. | Jin XH 8967 (PE) | KF852755 | KF852710 | KF852635 | KF852667 |
| *T. viridifusca* (Hook.) Benth. & Hook.f. | Jin XH 13608 (PE) | KF852757 | KF852712 | - | KF852669 |
| **Outgroups** |  |  |  |  |  |
| *Cybidium eburneum* Lindl. | unknown/unknown/NYBG-living 4527 | AB519784^30^ | AF448863^31^ | AY380983^3^ | - |
| *Earina autumnalis* (G.Forst.) Hook. f. | Chase O-298 (K)/Heidelberg BG 12446 (unknown)/Cameron 9810 (NY)/- | AF074155^1^ | EF079336^6^ | AY380993^3^ | - |
| *Epidendrum campestre* Lindl. | Borba EL 553 (UEC)/unknown/NYBG-living 2017/- | AF518060^7^ | AF263781^2^ | AY380996^3^ | - |

Note: One voucher represent all sequences from the same specimen.

**Table S3 Primers used for amplification and sequencing in this study**

| Loci | Name | Sequence (5’-3’) | Reference |
| --- | --- | --- | --- |
| *rbcL* | 1F | ATG TCA CCA CAA ACA GAA AC | Goldman et al., 2001^2^ |
|  | 1360R | CTT CAC AAG CAG CAG CTA GTT C | Goldman et al., 2001^2^ |
| *matK* | 19F | CGT TCT GAC CAT ATT GCA CTA TG | Kocyan et al., 2004^14^ |
|  | 390F | CGA TCT ATT CAT TCA ATA TTT C | Cuénoud et al., 2002^34^ |
|  | trnK-2R | AAC TAG TCG GAT GGA GTA G | Hidayat et al., 2005^35^ |
| *psaB* | NY159 | ACG CGT CGT ATT TGG TTT GGT ATT GC | Cameron et al., 2004^3^ |
|  | NY160 | CAA TGC CAA TAA AAA GTA ACC CAT CC | Cameron et al., 2004^3^ |
| *trnH*-*psbA* | trnH | CGC GCA TGG TGG ATT CAC AAT CC | Tate & Simpson, 2003^36^ |
|  | psbA | GTT ATG CAT GAA CGT AAT GCT C | Sang et al., 1997^37^ |

**Table S4 Partition analysis of Bayesian inference**

| Partition strategy | Identity |
| --- | --- |
| Subfamily-wide matrix |  |
| P1 | all data combined |
| P2 | coding gene combined; *matK* |
| P3 | *rbcL*; *psaB*; *matK* |
| P5 | coding genes codon 1st+2nd combined; coding genes code 3rd combined; *matK* |
| Collabieae matrix |  |
| P1 | all data combined |
| P3 | coding genes combined; *matK*; *trnH*-*psbA* |
| P4 | *rbcL*; *psaB*; *matK*; *trnH-psbA* |
| P6 | coding genes codon 1st+2nd combined; coding genes code 3rd combined; *matK*; *trnH*-*psbA* |

**Table S5 Morphological data matrix for the phylogenetic analysis**

| Taxa | Characters |
| --- | --- |
|  | 12345678901234567890123456789012345678901 |
| *Acanthephippium mantinianum* | 00110111100111111001120122110110211101102 |
| *Ancistrochilus rothschildianus* | 00110110100111110001120102111110211101002 |
| *Calanthe alleizettei* | 00110110101011110000120121111100201101100 |
| *Calanthe alpina* | 00110110101011110000120121111100201101100 |
| *Calanthe clavata* | 00110110100011110000120121111100211101110 |
| *Calanthe delavayi* | 00110110100011110000120121111100211101101 |
| *Calanthe davidii* | 00110110100011110000120121111100211101100 |
| *Calanthe densiflora* | 00110110100011110000120121111100211101110 |
| *Calanthe labrosa* | 00110110100011110000120121111100211101110 |
| *Calanthe mannii* | 00110110100011110000120121111100201101100 |
| *Calanthe triplicata* | 00110110100011110000120121111100211101100 |
| *Calanthe reflexa* | 00110110100011110000120122111100211101100 |
| *Calanthe tricarinata* | 00110110100011110000120122111100211101100 |
| *Calanthe vestita* | 001100?0100111110000120121?10100211110100 |
| *Cephalantheropsis gracilis* | 00110110100011111000120122011110210101112 |
| *Chrysoglossum ornatum* | 00110101100111110000100100111100001111112 |
| *Chrysoglossum assamicum* | 00111101100111110000100100111100201111112 |
| *Chrysoglossum latifolium* | 00?11101100111110000100100111100000111112 |
| *Collabium chinense* | 00?11111100111110000100101111100000111112 |
| *Collabium formosanum* | 00111111100111110000100101111100000111112 |
| *Collabium simplex* | 00111111100111110000100101111100000111112 |
| *Diglyphosa latifolium* | 00111101100111110000100101111100001111112 |
| *Eriodes barbata* | 00110111100111110000120111011102011101112 |
| *Gastrorchis pulchra* | 00110110100011110000120102101112010111112 |
| *Hancockia unifolia* | 00111100100111111000120111111110200111112 |
| *Ipsea* sp. | 00111111100111111001120110111111210010112 |
| *Mischobulbum papuanum* | 00111100100111111001120120101110201110112 |
| *Nephelaphyllum pulchrum* | 00?11110100011111001120101101100200010112 |
| *Nephelaphyllum tenuiflorum* | 00111100100111111001120101101100200100112 |
| *Phaius tankervilleae* | 00110111100011110000120101111100211101102 |
| *Phaius hainanensis* | 00110111100011110000120101111100201111102 |
| *Phaius minor* | 00110111100011110000120101111100201111102 |
| *Phaius mishmensis* | 00110111100011110000120101111100211101102 |
| *Phaius flavus* | 00110111100011110000120101111100211101102 |
| *Risleya purpurea* | 00?0?0?0100011?00100110002?00101?1?0?0102 |
| *Spathoglottis pacifica* | 00110101100011110000120112111110211101102 |
| *Spathoglottis plicata* | 00110101100011110000120122111110201111102 |
| *Tainia dunnii* | 00?11101100111110001120100111110201111112 |
| *Tainia latifolia* | 00?11111100111110001120100111110201111112 |
| *Tainia minor* | 00?11101100111110001120100111110201111112 |
| *Tainia hookeriana* | 00?10111100111110000120101111110201111102 |
| *Tainia viridifusca* | 00?10111100111110000120101111110201111102 |
| Outgroup |  |
| *Cymbidium eburneum* | 10110111000111110100100122110100001111002 |
| *Earina autumnalis* | 1011011010001111??001101?2111?10101111002 |
| *Epidendrum campestre* | 10110110100011110010100112111110001111002 |

**Note: morphological characters analyzed**

1. root epidermis 0=rhizodermis, 1=velamen

2. growth pattern 0=sympodial, 1=monopodial

3. phyllotaxy 0=spiral, 1=distichous

4. thickened stem 0=absent, 1=present

5. number of thickened stem internodes 0=several, 1=one

6. winter leaf 0=absent, 1=present

7. leaf articulation 0=absent, 1=present

8. inflorescence position 0=terminal, 1=lateral

9 perianth abscission 0=present, 1=absent;

10. slipper-shaped labellum 0=absent, 1=present

11. apiculate sepals 0=absent, 1=present

12 column foot 0=absent, 1=present

13. median outer stamen 0= absent, 1= present

14. lateral inner stamens 0=present, 1=absent

15. anther orientation 0=erect, 1=bending late

16. operculate anther 0=absent, 1=present

17. basal caudicle 0=absent, 1=present

18. stipe 0=absent, 1=present

19. massulae 0=absent, 1=epidendroid

20. pollinium of equal size 0=present, 1=absent

21. pollinium texture 0=granular, 1=solid

22. pollinium number 0=2, 1=4, 2=8

23. pollinium orientation 0=juxtaposed, 1=superposed

24. stigma 0=protruded, 1=sunken

25. viscidium 0=none, 1=diffuse, 2=detachable

26. lip base 0=saccate, 1=spurred, 2=neither saccate nor spurred

27. lip appendages 0=absent, 1=present,

28. lip claw 0=absent, 1=present

29. rhizome 0=absent, 1=present

30. column wings 0=absent, 1=present

31. rostellum 0=not developed, 1=protruding,

32. autotrophy, 0= present, 1= absent

33. leaf morphology 0=plicate, 1=conduplicate,

34. tuber 0=absent, 1=present

35. leaf number 0=one on each tuber or pseudobulb, 1=two or more

36. pollinia in separate pairs 0=absent, 1=present;

37. leaf petiole 0=absent, 1=present

38. flower non-resupinate 0=present, 1=absent

39. habit 0= epiphtyic, 1= terrestrial

40. elongate rhizome 0= absent, 1=present

41. column united with lip, 0=united to tip, 1=united but not to tip, 2=not united

References

1. Cameron KM, Chase MW, Whitten WM, Kores PJ, Jarrell DC, et al. (1999) A phylogenetic analysis of the Orchidaceae: evidence from *rbcL* nucleotide. American Journal of Botany 86: 208-224.

2. Goldman DH, Freudenstein JV, Kores PJ, Molvray M, Jarrell DC, et al. (2001) Phylogenetics of Arethuseae (Orchidaceae) based on plastid *matK* and *rbcL* sequences. Systematic Biology 26: 670-695.

3. Cameron KM (2004) Utility of plastid *psaB* gene sequences for investigating intrafamilial relationships within Orchidaceae. Molecular Phylogenetics and Evolution 31: 1157-1180.

4. Freudenstein JV, van den Berg C, Goldman DH, Kores PJ, Molvray M, et al. (2004) An expanded plastid DNA phylogeny of Orchidaceae and analysis of jackknife branch support strategy. American Journal of Botany 91: 149-157.

5. Pridgeon AM, Solano R, Chase MW (2001) Phylogenetic relationships in Pleurothallidinae (Orchidaceae): combined evidence from nuclear and plastid DNA sequences. American Journal of Botany 88: 2286-2308.

6. Gorniak M, Szlachetko DL. Phylogenetic relationships of the Orchidales based on molecular data. (Unpublished, data from NCBI).

7. van den Berg C, Goldman DH, Freudenstein JV, Pridgeon AM,Cameron KM, et al.(2005) An overview of the phylogenetic relationships within Epidendroideae inferred from multiple DNA regions and recircumscription of Epidendreae and Arethuseae (Orchidaceae). American Journal of Botany 92: 613-624.

8. van den Berg C. Molecular phylogenetics of Epidendreae (Orchidaceae). (Unpublished, data from NCBI).

9. Sierra SE, Gravendeel CB, de Vogel EF (2000) Revision of *Coelogyne* section *Verrucosae* (Orchidaceae): a new sectional delimitation based on morphological & molecular evidence. Blumea 45: 275-318.

10. Yukawa T, Ohba H, Cameron KM, Chase MW (1996) Chloroplast DNA phylogeny of subtribe Dendrobiinae

(Orchidaceae): Insights from a combined analysis based on *rbcL* sequences and restriction site variation. Journal of Plant Research 109: 169-176.

11. Freudenstein JV, Senyo DM (2008) Relationships and evolution of *matK* in a group of leafless orchids (*Corallorhiza* and Corallorhizinae; Orchidaceae: Epidendroideae). American Journal of Botany 95: 498-505.

12. Xiang XG, Li DZ, Jin WT, Zhou HL, Li JW, Jin XH (2012) Phylogenetic placement of the enigmatic orchid genera *Thaia* and *Tangtsinia*: Evidence from molecular and morphological characters. Taxon 61: 45-54.

13. Gravendeel B, Chase MW, de Vogel EF, Roos MC, Mes TH, Bachmann K (2001) Molecular phylogeny of *Coelogyne* (Epidendroideae; Orchidaceae) based on plastid RFLPS, *matK*, and nuclear ribosomal ITS sequences: evidence for polyphyly. American Journal of Botany 88: 1915-1927.

14. Kocyan A, Qiu YL, Endress PK, Conti E (2004) A phylogenetic analysis of Apostasioideae (Orchidaceae) based on ITS, *trnL*-*F* and *matK* sequences. Plant Systematics and Evolution 247: 203-213.

15. Whitten WM, Neubig KM, Williams NH. Molecular phylogenetics of Oncidiinae (Orchidaceae). (Unpublished, data from NCBI).

16. Chase MW, Williams NH, de Faria AD, Neubig KM, Amaral Mdo C, Whitten WM (2009) Floral convergence in Oncidiinae (Cymbidieae; Orchidaceae): an expanded concept of *Gomesa* and a new genus *Nohawilliamsia*. Annals of Botany 104: 387-402.

17. van den Berg C, Ryan A, Cribb PJ, Chase MW (2002) Molecular phylogenetics of *Cymbidium* (Orchidaceae: Maxillarieae): sequence data from internal transcribed spacers (ITS) of nuclear ribosomal DNA and plastid *matK*. Lindleyana 17: 102-111.

18. Parveen I, Singh HK, Raghuvanshi S, Babbar SB. DNA barcoding of orchids. (Unpublished, data from NCBI).

19. Roy M, Watthana S, Stier A, Richard F, Vessabutr S, Selosse MA (2009) Two mycoheterotrophic orchids from Thailand tropical dipterocarpacean forests associate with a broad diversity of ectomycorrhizal fungi. BMC Biology 7: 51.

20. Tobe H, Shinohara W, Utani N, Wiriadinata H, Girmansyah D, et al. Inventory studies of plant diversity on Lombok Island in Indonesia: An approach using DNA barcodes. (Unpublished, data from NCBI).

21. Cameron KM (2005) Leave it to the leaves: a molecular phylogenetic study of Malaxideae (Epidendroideae, Orchidaceae). American Journal of Botany 92: 1025-1032.

22. Whitten WM, Williams NH, Chase MW (2000) Subtribal and generic relationships of Maxillarieae (Orchidaceae) with emphasis on Stanhopeinae: combined molecular evidence. American Journal of Botany 87: 1842-1856.

23. Subedi A, Vermeulen JJ, Chaudhary RP, Jin XH, Gravendeel B. Taxonomic revision of *Coelogyne* sect. *Ocellatae* (Orchidaceae). (Unpublished, data from NCBI).

24. Kores PJ, Molvray M, Weston PH, Hopper SD, Brown AP, et al. (2001) A phylogenetic analysis of Diurideae (Orchidaceae) based on plastid DNA sequence data. American Journal of Botany 88: 1903-1914.

25. Pridgeon AM, Solano R, Chase MW (2001) Phylogenetic relationships in Pleurothallidinae (Orchidaceae): combined evidence from nuclear and plastid DNA sequences. American Journal of Botany 88: 2286-2308.

26. Bytebier B, Bellstedt DU, Linder HP (2007) A molecular phylogeny for the large African orchid genus *Disa*. Molecular Phylogenetics and Evolution 43: 75-90.

27. Salazar GA, Chase MW, Soto Arenas MA, Ingrouille M (2003) Phylogenetics of Cranichideae with emphasis on Spiranthinae (Orchidaceae, Orchidoideae): evidence from plastid and nuclear DNA sequences. American Journal of Botany 90: 777-795.

28. Alvarez-Molina A, Cameron KM (2009) Molecular phylogenetics of Prescottiinae s.l. and their close allies (Orchidaceae, Cranichideae) inferred from plastid and nuclear ribosomal DNA sequences. American Journal of Botany 96: 1020-1040.

29. Whitten WM, Damian Loayza M, Williams NH (2005) *Phragmipedium kovachii*: Molecular systematics of a New World Orchid. Orchids 74: 132-137.

30. Asahina H, Shinozaki J, Masuda K, Morimitsu Y, Satake M. Phylogenetic analyses using *matK* and *rbcL* sequences for identification of medicinal *Dendrobium* species. (Unpublished, data from NCBI).

31. Teng YF, Wu XJ, Wang ZT, Yu GD. Molecular phylogeny of *Dendrobium* based on *matK* gene. (Unpublished, data from NCBI).

32. van der Berg C. Molecular phylogenetics of Laeliinae based on *matK*, *trnL-F* and ITS DNA sequences. (Unpublished, data from NCBI).

33. Kocyan A, de Vogal EF, Conti E, Gravendeel B. 2008. Molecular phylogeny of *Aerides* (Orchidaceae) based on one nuclear and two plastid markers: A step forward in understanding the evolution of the Aeridinae. 48: 422-443.

34. Cuénoud P, Savolainen V, Chatrou LW, Powell M, Grayer RJ, et al. (2002) Molecular phylogenetics of Caryophyllales based on nuclear 18S rDNA and plastid *rbcL*, *atpB* and *matK* DNA sequences. American Journal of Botany 89: 132-144.

35. Hidayat T, Yukawa T, Ito M (2005) Molecular phylogenetics of subtribe Aeridinae (Orchidaceae): insights from plastid *matK* and nuclear ribosomal ITS sequences. Journal of Plant Research 118: 271-284.

36. Sang T, Crawford DJ, Stuessy TF (1997) Chloroplast DNA phylogeny, reticulate evolution, and biogeography of *Paeonia* (Paeoniaceae). American Journal of Botany 84: 1120-1136.

37. Tate JA, Simpson BB (2003) Paraphyly of *Tarasa* (Malvaceae) and diverse origins of the polyploid species. Systematic Botany 28: 723-737.
